# The type III secretion system facilitates systemic infections of *Pseudomonas aeruginosa* in the clinic

**DOI:** 10.1128/spectrum.02224-23

**Published:** 2023-12-13

**Authors:** Tiantian Wu, Zhenchuan Zhang, Tong Li, Xu Dong, Dan Wu, Lixia Zhu, Kaijin Xu, Ying Zhang

**Affiliations:** 1 Department of Infectious Diseases, State Key Laboratory for Diagnosis and Treatment of Infectious Diseases, National Clinical Research Center for Infectious Diseases, The First Affiliated Hospital, Zhejiang University School of Medicine, Hangzhou, China; 2 Research Center for Healthcare Data Science, Zhejiang Lab, Hangzhou, China; 3 Research and Service Center, College of Pharmaceutical Sciences, Zhejiang University, Hangzhou, China; 4 College of Food and Health, Zhejiang A&F University, Lin'an, Hangzhou, China; 5 Department of Hematology, The First Affiliated Hospital, Zhejiang University School of Medicine, Hangzhou, China; 6 Jinan Microecological Biomedicine Shandong Laboratory, Jinan, China; Universidad Andres Bello, Santiago, Chile

**Keywords:** T3SS, *Pseudomonas aeruginosa*, virulence, exoU, ST463/O4, high-risk clone

## Abstract

*Pseudomonas aeruginosa* is a major opportunistic pathogen that can cause severe systemic infections. Here, we found that the type III secretion system effector ExoU was the main determinant of pathogenicity of a highly virulent clinical isolate strain, BSI_S5, of *P. aeruginosa*, which caused severe bloodstream infections and belongs to ST463/O4. Deletion of *exoU* showed significantly attenuated cytotoxicity and virulence *in vivo*, while deletion of two other unique genes from strain BSI_S5, *chr_1696* and *chr_4238*, which encodes type I secretion system permease/ATPase or polysaccharide biosynthesis protein, respectively, caused no apparent loss of toxicity compared to the parent strain, and complementation of the *ΔexoU* mutant restored its virulence. The presence of ExoU-induced lung lesions and the mucosal damage of gallbladder in mice, and the replication of the *ΔexoU* mutant was hindered *in vivo* compared with the parent strain. We show here for the first time that a clinical isolate with intact *exoU* harbored a mutation of T→G substitution in the adjacent specific Pseudomonas chaperone for ExoU (*spcU*), which caused phenylalanine to valine change at amino acid 94 of SpcU, resulting in significant loss of cytotoxicity. This finding suggests that intact *exoU* is not sufficient for cytotoxicity, but a functional downstream SpcU is required for ExoU secretion and cytotoxicity. Finally, we found that BSI_S5 harbored two types of insertion sequences adjacent to *exoU*, suggesting the potential of *exoU* to be transferred to other strains. Our study opens an avenue for further mechanistic study of *P. aeruginosa* systemic infections regarding its high pathogenicity and clinical spread.

**IMPORTANCE:**

The identification of decisive virulence-associated genes in highly pathogenic *P. aeruginosa* isolates in the clinic is essential for diagnosis and the start of appropriate treatment. Over the past decades, *P. aeruginosa* ST463 has spread rapidly in East China and is highly resistant to β-lactams. Given the poor clinical outcome caused by this phenotype, detailed information regarding its decisive virulence genes and factors affecting virulence expression needs to be deciphered. Here, we demonstrate that the T3SS effector ExoU has toxic effects on mammalian cells and is required for virulence in the murine bloodstream infection model. Moreover, a functional downstream SpcU is required for ExoU secretion and cytotoxicity. This work highlights the potential role of ExoU in the pathogenesis of disease and provides a new perspective for further research on the development of new antimicrobials with antivirulence ability.

## INTRODUCTION

*Pseudomonas aeruginosa* is a Gram-negative bacterial pathogen that could reside and replicate within a host and cause diverse infections, especially in immunocompromised hosts (1), such as nosocomial bacteremia, ventilator-associated pneumonia, cystic fibrosis (CF) and chronic obstructive pulmonary disease (COPD) (2 – 4). Although increasing multidrug resistance continues to complicate treatment (5), the pathogenicity of *P. aeruginosa* appears to be related to its diverse virulence factors, including lipopolysaccharide (LPS), outer membrane proteins, biofilm formation (flagellum, pili, and other adhesins), secretion systems [type I secretion system (T1SS) to T6SS], and exopolysaccharides (6). The repertoire of genes facilitating its survival and virulence in hosts is highly flexible, showing a large variety among natural isolates thriving in different environments (7).

One widely studied and important virulence determinant of *P. aeruginosa* is its type III secretion system (T3SS). T3SSs are multiprotein complexes that form syringe-like structures on the surface of bacterial cells, allowing effector proteins to be delivered directly from the bacteria into the cytoplasm of the host cell (8). There are four major toxic effectors (ExoU, ExoT, ExoS, and ExoY) injected via T3SS that are un-uniformly distributed in clinical isolates of *P. aeruginosa*. Despite being produced by less than half of clinical isolates (9), ExoU, a potent phospholipase, is considered the major T3SS cytotoxin because it has the greatest impact on disease severity, being associated with severe acute lung injury, sepsis, and a relevant biomarker of early mortality in *P. aeruginosa* bacteremia (10 – 13).

Meanwhile, previous studies have examined the genetic diversity of *P. aeruginosa* clinical isolates. Several “high-risk” clones of *P. aeruginosa* defined by multilocus sequence typing (MLST) have spread worldwide and are frequently associated with epidemics where multidrug resistance confounds treatment, including serotypes ST111, ST175, ST235, and ST395 (14). Over the past few years, ST235 has mostly emerged as a global clone due to a unique combination of virulence genes (*exoU* and a *dprA*-related gene) and high-level antibiotic resistance (15). In China, the surveillance of carbapenem-resistant *P. aeruginosa* (CRPA) from 2006 to 2018 showed that ST463 has become dominant and is highly resistant to β-lactams, including carbapenems and fluoroquinolones. The emergence and expansion of ST463 are associated with plasmid-borne *blaKPC-2* and virulence-related genes (16). The surveillance of KPC-producing *P. aeruginosa* isolates from different geographic regions in China showed that ST463 (*n* = 107, 70.9%) was the main CRPA clone in East China (17). A recent study reported that ST463 *P. aeruginosa* is closely associated with *blaKPC-2* and *exoU* and is spreading rapidly (18). A retrospective cohort study demonstrated that the mortality rates of the ST463 CRPA bloodstream infections (BSIs) were higher than those in the non-ST463 CRPA BSI, and the emergence and spread of the ST463 CRPA clone harboring the *exoU* gene in East China pose a new threat in the clinic (19). However, previous investigations mainly examined the prevalence and potential pathogenicity of various type III secretory proteins and correlated these phenotypes with cytotoxicity or clinical outcomes in human *P. aeruginosa* infections, and their role in the pathogenicity of *P. aeruginosa* has not been fully characterized. Further detailed characterization of virulence determinants and especially type III secretory proteins would shed light on how the virulence genes are expressed and regulated from the clinically relevant *P. aeruginosa*.

This study analyzed a batch of clinical isolates of *P. aeruginosa* from different clinical conditions, and one strain in particular caught our attention, which we named *P. aeruginosa* BSI_S5 (S5), obtained from the bloodstream infection of a patient diagnosed with acute myeloid leukemia with poor prognosis. Given the poor outcome of the patient and the fact that the strain showed resistance to both β-lactams and fluoroquinolones, we sought to explain the causes that may explain the poor clinical outcome. Firstly, to identify virulence genes of *P. aeruginosa* BSI_S5, we determined the complete genome sequence of five selected clinical strains of *P. aeruginosa* from different conditions along with PAO1 as the reference strain and performed a comparative genomic analysis to identify specific genes that are present in the highly virulent clinical isolate BSI_S5 but absent in low virulence strains, followed by knockout of candidate virulence genes and virulence screens. Using this approach*,* we identified *exoU* as the main driving force that caused high virulence of the *P. aeruginosa* BSI_S5 strain belonging to the ST463 serotype in different hosts at the molecular level.

## RESULTS

### Virulence of *P. aeruginosa* isolates in chick embryo model and cytotoxicity assay

**Fig 1:**
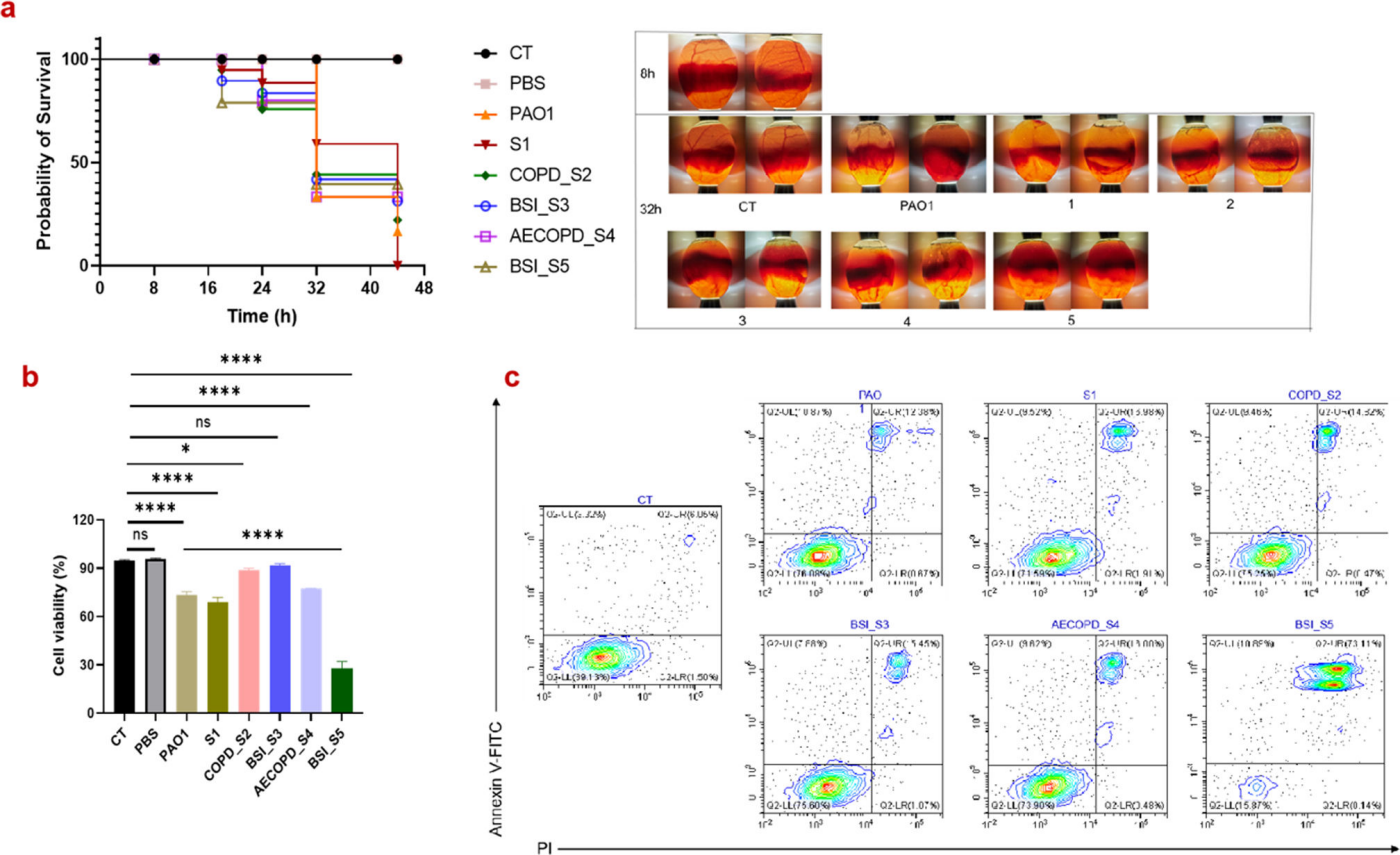
BSI_S5 was the most virulent strain among the six *P. aeruginosa* strains.The Kaplan–Meier chick embryo survival curve and morphological changes in chick embryos (10 days of age) at different times (**a**). Cell viability of THP-1 challenged with different *P. aeruginosa* (**b**). Representative flow cytometry analysis of THP-1 apoptosis (**c**). Data are presented as mean ± standard deviation of at least three biological replicates. ^ns^ *P* >0.05, *P <  0.05, ****P  <  0.0001 (one way analysis of variance); and fluorescence activated cell sorting (FACS) of infected THP-1 (3.5 hpi, multiplicity of infection = 5:1). CT, uninfected control.

The chicken embryo could be served as a reliable, inexpensive, and easy setup for assessing bacterial virulence, and it has been used for different bacterial species including *Listeria monocytogenes*, *Salmonella* Typhimurium, *Staphylococcus aureus*, *Escherichia coli*, *Enterococcus cecorum*, and *P. aeruginosa*. (20 – 27). We used the chicken embryo lethality assay to assess the pathogenic potential of different clinical strains of *P. aeruginosa* with PAO1 as a reference strain. As shown in Fig. 1a, the survival rates at 18th hour post-infection (hpi) were lower in embryos infected with strain BSI_S5 (60%), BSI_S3 (80%), and S1 and COPD_S2 (both 90%) than those infected with AECOPD_S4 and PAO1 (both 100% survival). Among the *P. aeruginosa* clinical strains, the survival rate on the 32nd hpi was the lowest for S4 and S5 (0%), demonstrating the highest embryo mortality and shortest survival. Finally, infected embryos all died at 44 hpi when a dose of 10^5^ CFU of different strains of *P. aeruginosa* was applied.

We also compared the morphological features of the infected chicken embryos, and the results showed that the infected embryos were identified by the absence of spontaneous movement, usually associated with hemorrhage, and the small black dot at the other end of the air chamber, loss and blackening of vascular architecture, and abnormal morphology. The loss and blackening of vascular architecture were the most severe for embryos infected by BSI_S5 on the 32nd hpi.

To further evaluate the cytotoxicity of the clinical strains of *P. aeruginosa*, we used a commonly adopted cell infection model with THP-1 cells. The infected THP-1 cells had 28.09% ± 4.06% viability at 3.5 hpi with BSI_S5, which was significantly higher than all the other groups (*P* < 0.0001). The value for PAO1, S1 or AECOPD_S4 was 73.22% ± 2.28%, 68.79% ± 3.14%, and 77.56% ± 0.13%, respectively, and these values were significantly higher than uninfected cells [uninfected control (CT) or phosphate-buffered saline (PBS)] (*P* < 0.0001). The cell viability for THP-1 cells infected with COPD_S2 and BSI_S3 was 89.00% ± 0.97% and 91.68% ± 1.20%, respectively, demonstrating their low cytotoxicity (Fig. 1b). Also, we used the annexin V-FITC/PI (fluorescein isothiocyanate/propidium iodide) to determine if there was externalization of phosphatidylserine residues on the outer plasma membrane of apoptotic cells. THP-1 cells infected with BSI_S5 showed the highest proportion of double staining by annexin V-FITC/PI, which indicates apoptotic and necrotic cell death (Fig. 1c). Taken together, these results demonstrated that S5 was the most virulent among the strains. S5 was isolated from the blood sample of a 53-year-old female patient hospitalized with acute myeloid leukemia complicated with bilateral lung and biliary infections. It was isolated as the causative pathogen of systemic infections (it also existed in urine and sputum). The clinical background and bacterial isolates used are listed in Table S1 through S2.

### Features of *P. aeruginosa* genomes

**Fig 2:**
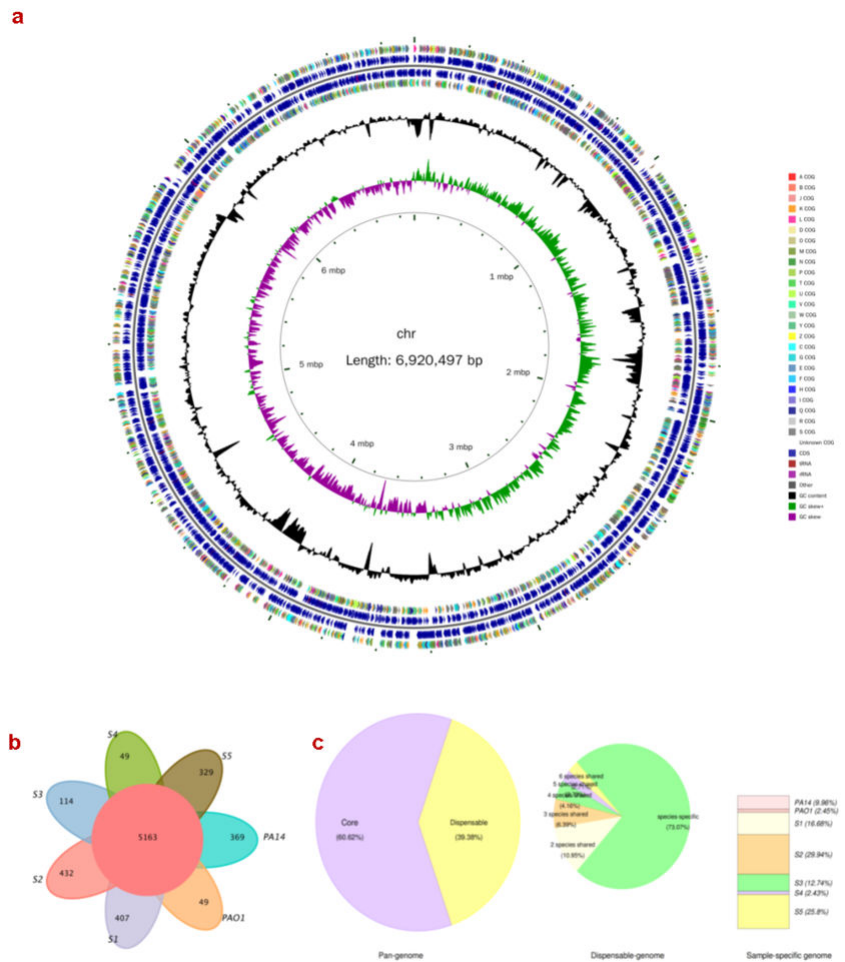
Circular representation and gene family analysis of the *P. aeruginosa* genome.Circular representation of the *P. aeruginosa* BSI_S5 genome. From inside out, the innermost circle indicates the chromosomal location in base pairs; the second circle represents the GC sketch; the third circle represents the G + C content; circles 4 and 7 show the classification of CDS into functional categories based on clusters of orthologous groups of proteins; circles 5 and 6 show the location of CDS, tRNAs, or rRNAs on each chromosome in blue (**a**). Gene family analysis of PA14, PAO1, S1, COPD_S2 (S2), BSI_S3 (S3), AECOPD_S4 (S4), and BSI_S5 (S5). Petal map of gene family of different strains of *P. aeruginosa* (**b**) and proportion distribution map of pan-genome and dispensable genome of *P. aeruginosa* (**c**).

To identify the molecular basis for the high-virulence strain BSI_S5, a hybrid assembly using PacBio sequencing as well as Illumina sequencing was performed to determine the complete genome sequence of the strain . The assembled genome of BSI_S5 has a single circular chromosome of 6,920,497 bp with an average GC content of 65.86% (Fig. 2a), which is larger than complete genomes of the reference strain *P. aeruginosa* PAO1 (6,268,251 bp) and other clinical isolates such as S1, BSI_S3, and AECOPD_S4, but smaller than COPD_S2 (7,181,513 bp) (Table 1). A sum of 6,252 genes were predicted from the S5 genome by the NCBI Prokaryotic Genome Annotation Pipeline server, which included 6,186 coding sequences and 67 tRNA genes and 16 rRNA operons and 79 ncRNA genes.

**TABLE 1:** Features of *P. aeruginosa* genomes

Strain	Chromosome (bp)	ORF^*a*^ number	GC (%)	Plasmid^*b*^
PAO1	6,268,251	5,683	66.5	/
S1	6,692,417	6,185	65.36	Plasmid 1: 53,494 bp; plasmid 2: 35,358 bp; plasmid 3: 52,415 bp
COPD_S2	7,181,513	6,624	65.97	/
BSI_S3	6,576,881	6,045	66.31	/
AECOPD_S4	6,316,174	5,718	66.48	/
BSI_S5	6,920,497	6,309	65.86	Plasmid 1: 170,019 bp; plasmid 2: 41,104 bp

^*a*^ ORF, open reading frame.

^*b*^ "/" represents no plasmid detected.

PA14, which was recognized as a virulent strain (28), was included as a control in the comparative genomics analysis. Comparison of the annotated genes in the *P. aeruginosa* PAO1 and PA14 genomes with those clinical *P. aeruginosa* genomes revealed an extensive conservation of a set of genes that are shared by all of the strains ( Fig. 2b and c). A total of 5,163 genes are conserved across all seven genomes analyzed, which are recognized as the *P. aeruginosa* core genome (60.62% of the whole genome). The genome for each strain carries a relatively modest number of unique sequences, where unique gene family numbers are 329, 49, 114, 432, 407, 49, and 369 for strain BSI_S5, AECOPD_S4, BSI_S3, COPD_S2, S1, PAO1, and PA14, respectively, confirming that *P. aeruginosa* harbors a large amount of strain-specific variations in protein-coding genes. Besides, there were three plasmids in S1 and two in BSI_S5, which can transfer horizontally between bacterial cells, within and between communities of the same or different species and play a crucial role in bacterial ecology and evolution (29).

### Virulence Factor Database analysis

**Fig 3:**
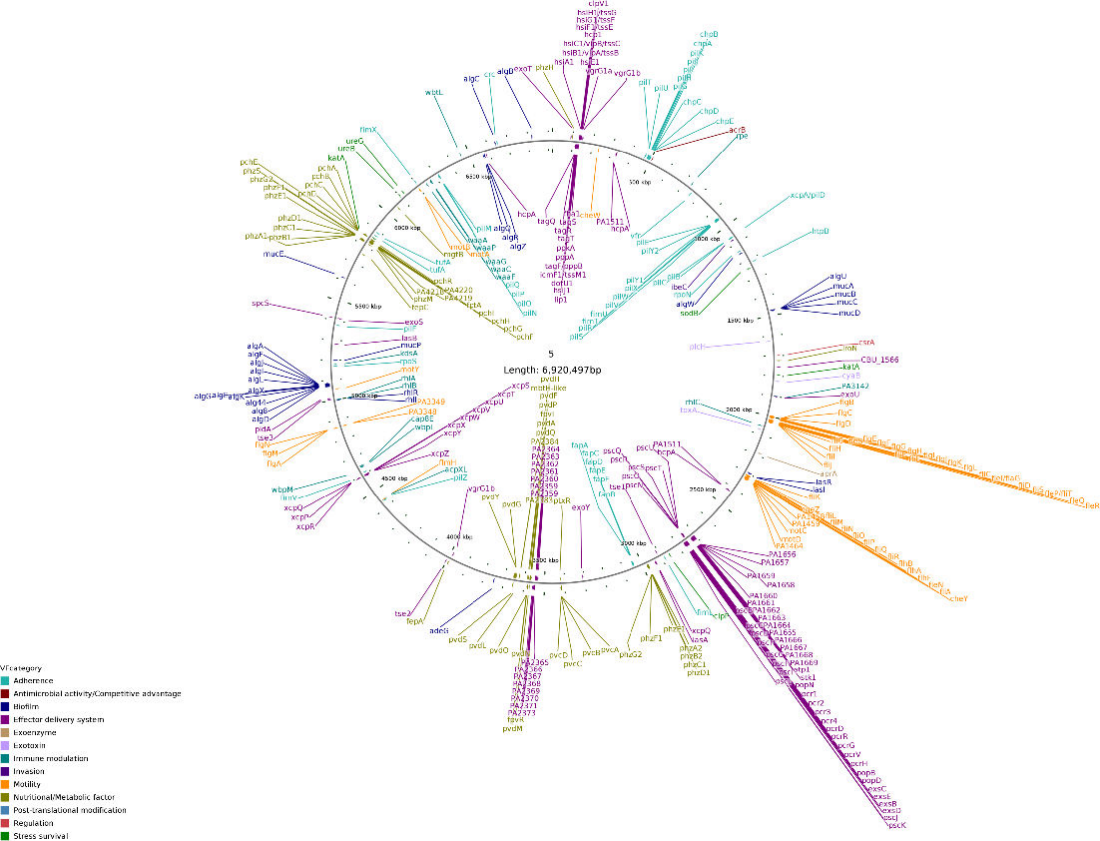
Location of virulence-associated genes in BSI_S5. A total of 442 virulence factors are identified in BSI_S5, and they belong to 13 different categories, which are marked in different colors.

The analysis and comparison of genomes showed a diversity of species-specific genes between strains. The distribution of virulence-associated genes was further analyzed among the core and accessory genome of these strains. Previous studies have identified several virulence factors in *P. aeruginosa*, including LPS, quorum sensing, two-component systems, type III secretion systems, outer membrane vesicles, CRISPR-Cas, and their regulation (6). According to the Virulence Factor Database analysis, there are eight major virulence factors in *P. aeruginosa* that include adherence (i.e., functional amyloid in *Pseudomonas*, type IV pili), effector delivery system [i.e., exolysin, HSI-2, HSI-2 T6SS secreted effectors, HSI-3, Hcp1 secretion island I, HSI-I T6SS secreted effectors, LasA, LasB (elastase), type III secretion system, TTSS secreted effectors, and xcp secretion system], motility (i.e., flagella), exotoxin (i.e., exotoxin A and phospholipase C), exoenzyme (i.e., alkaline protease), immune modulation (i.e., LPS and rhamnolipid), biofilm [i.e., alginate (mucoid exopolysaccharide), quorum sensing], and nutritional/metabolic factor (i.e., pyochelin, pyocyanin and pyoverdine). As for the most virulent strain S5 in this study, 442 genes related to the virulence were identified, and their locations are shown in Fig. 3. Apart from the general recognized virulence factors, some of these genes were related to antimicrobial activity/competitive advantages, invasion, post-translation modification, regulation, and stress survival. Three genes were unique to S5 among the six strains of *P. aeruginosa* analyzed in this study, which are located at chromosomes 1696, 1770, and 4238, respectively (Table 2 ).

**TABLE 2:** Unique virulence-related genes identified in strain BSI_S5

Contig ID	Types	Start	End	Strand	Gene name	Product
*chr_1696*	CDS	1,825,979	1,828,126	+		Type I secretion system permease/ATPase
*chr_1770*	CDS	1,900,395	1,902,458	+	*exoU*	Type III secretion system effector cytotoxin ExoU
*chr_4238*	CDS	4,475,984	4,477,018	−		Polysaccharide biosynthesis protein

As for the *chr_1696* (2,148 bp, type I secretion system permease/ATPase), BLAST alignment analysis showed its nucleotide sequence has 100% identity with the sequence in different strains of *P. aeruginosa*. Proteins encoded by *chr_1696* show a high level of amino acid homology to cyclolysin secretion ATP-binding protein.

The sequence of *chr_1770* (2,064 bp) showed 100% identity with *exoU*, which encodes a major virulence determinant of *P. aeruginosa*—T3SS effector cytotoxin ExoU. T3SS also injects other potent cytotoxins, including ExoS, ExoT, or ExoY, into eukaryotic cells. The production of each of the different enzymes determines a distinct host tissue injury, where ExoU is the one that has a greater impact on bacterial virulence (13) and has been associated with increased morbidity and mortality in patients with pneumonia and bacteremia (12, 13).

The nucleotide sequence of chr_4238 (1,035 bp) showed 100% identity with the sequence in different strains of *P. aeruginosa*. The protein encoded by *chr_4238* showed a high level of amino acid homology to type VIII capsular polysaccharide synthesis protein Cap8E or capsular polysaccharide biosynthesis protein Cps4J. Capsular polysaccharides are one of the major contributors to virulence of various microorganisms, as the presence of capsule enables these bacteria to escape from detection and clearance by the host immunity (30).

### *exoU* is critical for increased virulence in *P. aeruginosa*-induced infection

To identify which of the above three genes might play a role in determining the high virulence of *P. aeruginosa* BSI_S5, we first performed deletion mutant construction (Fig. S1) followed by complementation studies of the above-mentioned candidate genes. To determine if the deleted gene affected the growth of BSI_S5, their ability to grow in Luria-Bertani (LB) medium was tested by measuring the optical density at 600 nm. Results showed that there was no significant difference in growth curves between the parent strain and the deletion mutants (Fig. S2).

**Fig 4 F4:**
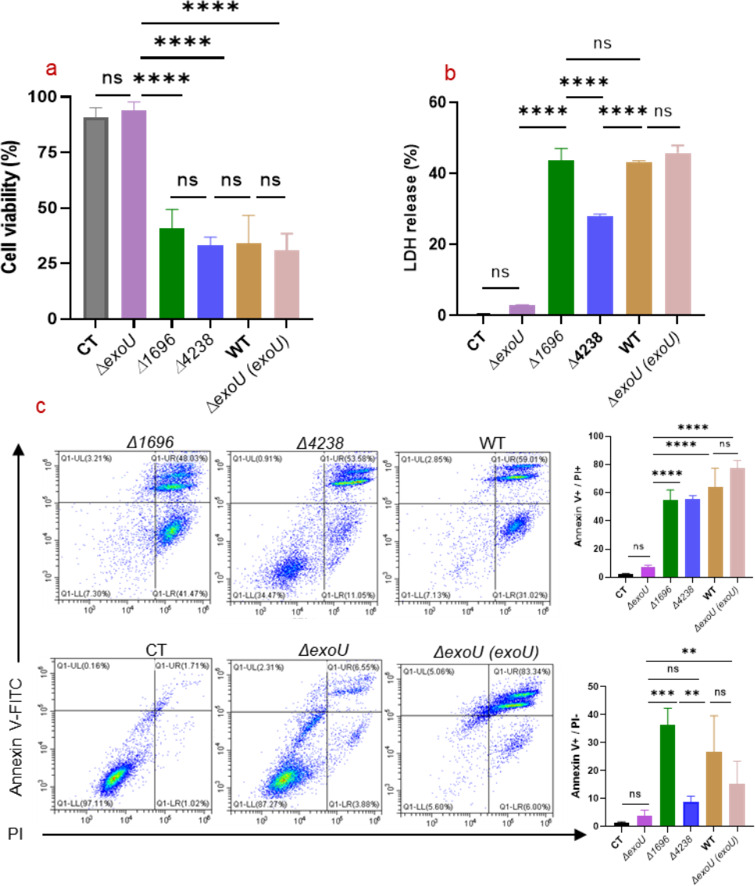
*exoU* is critical for increased virulence for THP-1 macrophages in *P. aeruginosa*-induced infection. Deletion of *exoU* showed attenuated cell toxicity (multiplicity of infection = 10:1) as indicated by trypan blue staining at 2.5 hpi (**a**). Lactate dehydrogenase assay of THP-1 infected by deletion strains and parent BSI_S5 at 2.5 hpi (**b**). Representative flow cytometry analysis of THP-1 apoptosis (**c**).WT*: P. aeruginosa* BSI_S5. *δexou* (*exoU*): BSI-S5 *δexou* complementation strain. Data are presented as mean ± standard deviation of at least three biological replicates. ^ns^ *P* > 0.05, **P  <  0.01, *** *P*  <  0.001, ****P  <  0.0001 (one-way analysis of variance). CT, uninfected control; WT, wild type.

Next, we adopted a widely used cytotoxicity model, which is based on evaluating the toxicity of the pathogen to THP-1 macrophages, to determine the virulence of the deletion mutants. Trypan blue staining showed the deletion of chr_*1696* and *chr_4238* caused no apparent loss of cytotoxicity, while deletion of *exoU* showed significantly decreased toxicity compared to the parent strain (*P <* 0.0001). In contrast, ∆*exoU* deletion mutation caused a dramatic loss of cytotoxicity with no significant difference between CT (*P* > 0.05), indicating severe attenuation of cytotoxicity for the *exoU* mutant (Fig. 4a).

**Fig 5 F5:**
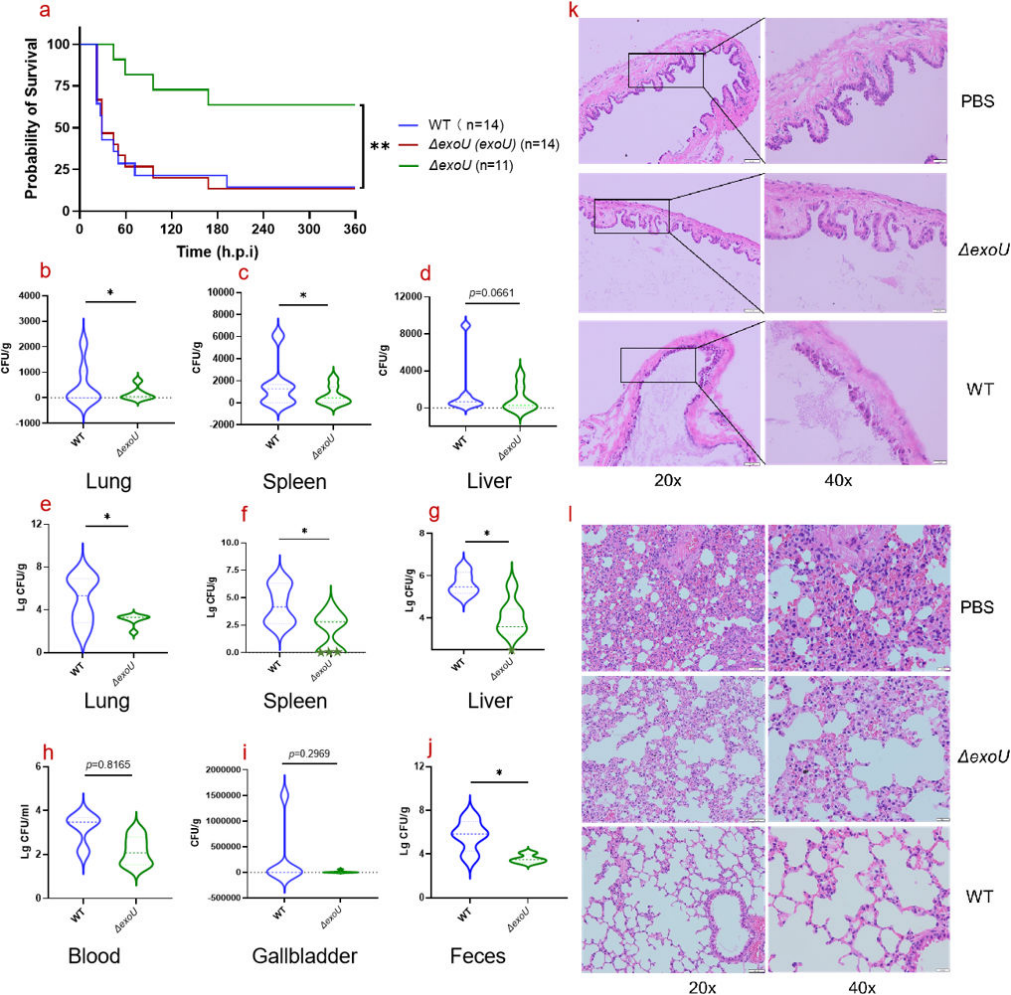
ExoU is critical for virulence and pathogenicity of *P. aeruginosa* infection.The Kaplan–Meier survival curve for C57BL/6 (7- to 9-week-old) female mice challenged with the *P. aeruginosa exoU* deletion mutant (*Δexou*, *n* = 11), *ΔexoU* complementation strain [*δexou (exoU)*, *n* = 14], or parent *P. aeruginosa* BSI_S5 (WT, *n* = 14) strain. Comparison of the survival curves was performed using log-rank (Mantel–Cox) test (**a**). Female BALB/c mice (*n*  =  7) were intravenously injected with ~5 × 10^6^ CFU of *P. aeruginosa* BSI_S5, *Δexou* (**b–d**). Female BALB/c mice (*n*  =  6) were intravenously injected with ~4 × 10^7^ CFU of WT, *δexou* (**e–j**). Mice were euthanized at 22  hpi (*n*  =  6), and bacteria were enumerated from several anatomical sites by plating serial dilutions of organ homogenates (CFU per gram of organ weight) and the recovered bacteria are presented (star on the *x*-axis means the number of CFU was below the detection limit (<10 CFU/g). Organs where no bacteria were recovered are denoted with a diamond on the *x*-axis. Lung (**b and e**), spleen (**c and f**), liver (**d and g**), blood (**h**), gallbladder (**i**), and feces (**j**). For input 1  g equals 1  mL of wet weight. GB and lung embedded in formalin were evaluated by hematoxylin and eosin staining (**k and l**). WT, wild type.

In addition, we used lactate dehydrogenase (LDH) assay to further confirm the cytotoxicity for the parent strain BSI_S5 and the deletion mutants. LDH assay is a common method for evaluating cytotoxicity, which is based on determining the activity of cytoplasmic enzymes released by damaged cells when cells undergo apoptosis, necrosis, and other forms of cellular damage (31). As shown in Fig. 4b, deletion of *exoU* showed attenuated cytotoxicity in the LDH assay, whose value was 2.99% ± 0.08% at 2.5 hPi, whereas the parent strain BSI_S5 induced 43.22% ± 0.46%. The polysaccharide biosynthesis protein mutant (*∆4238*) showed a decreased LDH value compared to the parent strain BSI_S5 (*P <* 0.0001), while the attenuation of *∆4238* mutant was less obvious when the infection time was increased to 4.5 hours (its virulence was not different from the parent strain at 4.5 hours) (*P* > 0.05) (Fig. S3a). However, no decrease in cytotoxicity was observed for the *∆1696* mutant even when infection time increased to 4.5 hours (Fig. S3a), suggesting the type I secretion system permease/ATPase does not contribute to the cytotoxicity.

As shown in Fig. 4c, the ratio of early apoptotic cells indicated by annexin V^+^/PI cells was significantly lower for the *ΔexoU*- and *Δ4238*-infected groups, while *Δ1696* and the parent strain BSI_S5 induced apoptosis at 36.34% ± 5.96 and 26.59% ± 13.02%, respectively. The percentage of the annexin V^+^/PI^+^-cells which represented late apoptotic and/or necrotic cells were 54.46% ± 7.30%, 55.34% ± 2.59%, and 64.33% ± 13.01% for *Δ1696*, *Δ4238*, and BSI_S5, respectively; these values were significantly higher than that for *ΔexoU* (7.50% ± 1.14%). Thus, deletion of *exoU* resulted in decreased cell apoptosis or necrosis. Meanwhile, complementation of *ΔexoU* restored virulence in the cytotoxicity assay (Fig. 4a through c).

### *exoU* contributes to *P. aeruginosa* virulence in mouse systemic infection *in vivo*

To further verify the role of ExoU during infection, *P. aeruginosa* BSI_S5 [parent strain, marked as wild type (WT)] and *ΔexoU* mutant were employed in a bloodstream infection utilizing a mouse model via tail vein. The results showed that the *ΔexoU* mutant-infected group had a 63.6% survival rate, versus only 14.3% (*P* < 0.05) for parent BSI_S5 over a period of 15-day observation. When the *exoU* gene was complemented in *trans* [*pK∆exoU* (*exoU*)], the survival rate of mice was comparable to that of WT (Fig. 5a). We also examined the fate of *P. aeruginosa* during the bloodstream infection to allow for consistent and reproducible delivery of bacteria directly into the bloodstream. Enumeration of bacterial counts from infected organs was conducted from mice infected with parent strain and *ΔexoU*. When each mouse was infected with a dose of 5 × 10^6^ CFU by tail-vein injection, *P. aeruginosa* was detected in the lungs, spleen, liver (Fig. 5b through d), and a minor proportion of blood (data not shown), but no *P. aeruginosa* spp. were detected in gallbladder (GB) and feces (data not shown) at 22 hpi. Bacterial recovery in the lung and spleen reached significantly higher numbers in the parent strain infection group than those for *ΔexoU* (*P* < 0.05), suggesting that *ΔexoU* replication was hindered *in vivo* compared with the parent strain.

When a high dose of 4 × 10^7^ CFU was applied, significant difference of bacterial recovery of parent strain was observed at 22 hpi in lungs, spleen, and liver, and a higher amount was detected in blood (Fig. 5e through h). Besides, a dramatic expansion of the bacterial population in the GB was observed for the majority (four of seven) of parent strain-infected group but only one of seven for *ΔexoU*-infected group (Figure 5i). Also, the dramatic expansion of the bacterial population was detected in feces for parent strain-infected mouse, which was significantly higher than *ΔexoU*-infected mouse (Fig. 5j).

We examined GBs of infected animals for histological evidence of injury. The wall of GBs from mock-infected mouse (control, PBS) consists of the mucosa, lamina propria, an irregular muscular layer, perimuscular connective tissue, and serosa (or visceral peritoneum) (32 – 34). The connective tissue along the hepatic surface is continuous with the interlobular connective tissue of the liver (33). In contrast, the mucosa of GBs from parent strain-infected mice displayed a general distortion of the cellular architecture characterized by disrupted lamina propria and damaged columnar epithelial cells. However, in contrast to the mucosal damage observed with parent strain infection, no damage to the GB epithelium was observed for *ΔexoU*-infected mice (Fig. 5k [Fig F4 F5] [Fig F5]). We then evaluated pulmonary lesions by histopathological analyses. Massive infiltration of inflammatory cells, destruction of alveolar wall structure, and detachment of epithelial cells were observed in the parent strain-infected group, whereas *ΔexoU* induced less detachment of epithelial cells compared with mice infected with parent strain.

The majority of previous studies mainly focused on the correlation between virulence and the *exoU* gene (10 – 13, 35). For example, *P. aeruginosa* strains isolated from hospital-acquired pneumonia patients were highly virulent if the isolate harbored the *exoU* gene compared with isolates that did not (36). Although *exoU* gene was known to be a major contributor to potential virulence of *P. aeruginosa* in terms of cytotoxicity (37, 38), in this study, we used comparative genomic analysis to identify the three unique genes, *chr_1696*, *chr 4238*, and *exoU*. By gene deletion and virulence screens, we finally confirmed that the *exoU* gene was the determinant affecting the virulence of *P. aeruginosa* BSI_S5 at the molecular level by gene deletion studies followed by virulence testing using cell and animal models, beyond a correlation analysis.

### Prevalence of *exoU* gene in clinical isolates of *P. aeruginosa*

Since our gene knockout and virulence analysis showed that only *exoU* was the main driving force that caused the high virulence of BSI_S5, we wanted to know if this gene is commonly present in clinical isolates of *P. aeruginosa* from different sources. To do so, we analyzed a panel of 72 respiratory or blood isolates of *P. aeruginosa* collected from different patients in The First Affiliated Hospital, Zhejiang University School of Medicine Zhejiang, Hangzhou, which means that the *exoU*+ genotype mainly occurred in the east of China in this study. In total, 8 (11.1%) of 72 examined clinical isolates harbored *exoU*-like sequences by polymerase chain reaction (PCR)-based gene detection assay. The detailed information of the *exoU*+ phenotype is listed in Table 3. Among the *exoU*+ strains, five strains were blood and sputum isolates; the remaining three were isolated from bile, interstitial fluid, or urine, respectively. The cytotoxicity assay indicated that the cell viability of THP-1 macrophages infected by seven of eight *exoU*+ strains showed more than 40% cell toxicity compared to CT (uninfected cells) at 2 hpi, indicating the acute cytotoxicity of these strains. However, it is interesting to note that an isolate named PA AP showed 2.97% ± 0.33% for cell toxicity at 2 hpi, which was not significantly higher than CT (*P* > 0.05), indicating its low and non-acute toxicity against THP-1 cells (Fig. S3b). Thus, although the presence of *exoU* is associated with high virulence, it is not always the case. To further identify the basis for lack of high virulence in the strain PA AP, whole-genome sequencing (WGS) of these eight isolates was conducted. The results confirmed the presence of *exoU* in the eight strains. Notably, half of them (four of eight) belonged to ST463, which showed high virulence, and the strain PA AP belonged to ST1682.

**TABLE 3:** The MLST genotype of *exoU*+ *P. aeruginosa* clinical isolates from patients^*a*^

Isolate	Sex	Age	Isolation year	Sample source	Primary diagnosis	MLST
PA UTI	Male	64	2023	Urine	Urinary tract infection	463
PA MODS1	Female	58	2021	Sputum	Multiple-organ dysfunction syndrome	New
PA RF1	Male	83	2021	Sputum	Respiratory failure	357
PA RC	Male	71	2021	Interstitial fluid	Rectal cancer	2069
PA W1	Male	74	2023	Sputum	Wound and renal Insufficiency	463
PA MDS1	Male	72	2021	Sputum	MDS	463
PA MDS2	Male	68	2022	Blood	MDS	463
PA AP	Male	98	2022	Blood	Abdominal pain	1682

^*a*^ MDS, myelodysplastic syndrome.

In this study, the presence of *exoU* was not specifically associated with any source of infection, which is in accord with the observation in a previous study (39). The distribution of the *exoU* gene seems to vary significantly; for example, the ratio was 13% (4 of 32) (40) or 64% (9 of 14) (41) of bacteremia isolates, 10% (3 of 29) of CF isolates (42), and 61.5% (8 of 13) of microbial keratitis isolates, whereas none of the CF isolates (0/9) possessed this gene (43), 12% (43 of 189) of clinical isolates (39), 28% (32 of 115) of clinical and environmental isolates (44), and 52% (83 of 160) of clinical isolates (100% in wound) (45). The variations in the prevalence of the *exoU+* strains among different studies could be ascribed to small numbers of samples analyzed or isolates not randomly selected or isolates from patients with different underlying clinical conditions with varied genotypes.

### Mutation in *spcU* downstream of *exoU* may affect ExoU secretion and cytotoxicity in a clinical isolate

**Fig 6:**
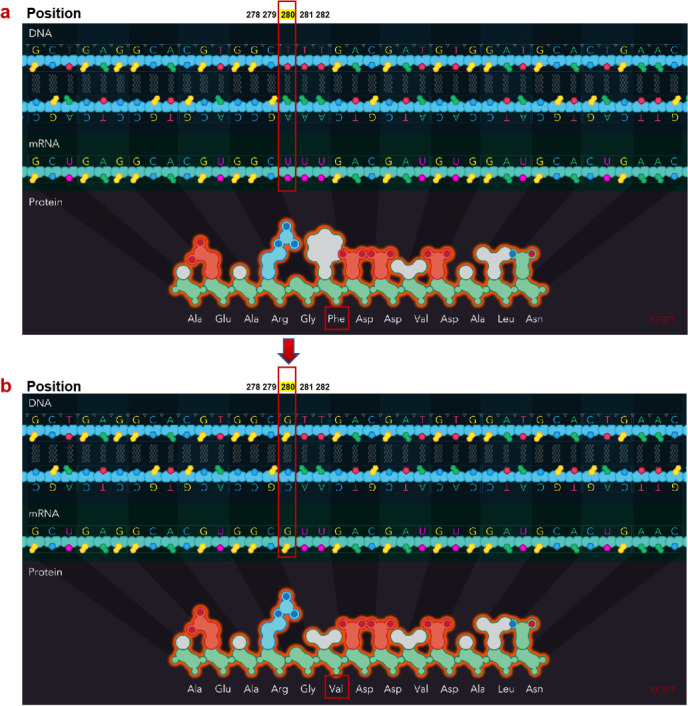
Demonstration of a point mutation in the *spcU* gene.The wild-type *spcU* gene sequence in *P. aeruginosa* BSI_S5 and the other seven *exoU+* clinical isolates with defined virulence (**a**). A point mutation of T→G at nucleotide position 280 in the *spcU* gene from the isolate named PA AP, causing phenylalanine to be replaced by valine at aa 94 of SpcU (SpcU-F94V) (**b**).

It is known that the increased virulence is associated with the secretion of ExoU, a toxin transported by the *P. aeruginosa* type III secretion system. However, not all strains of *P. aeruginosa* harboring the type III system (T3SS) genes are capable of secreting effector proteins (36). For example, 12 (34%) of the 35 examined *P. aeruginosa* isolates from patients with ventilator-associated pneumonia harbored the *exoU* gene, and immunoblot analyses showed that 83% of (10 of 12) strains produced detectable amounts of ExoU *in vitro* (35). Considering all the results together, we speculated that the strain PA AP showing low toxicity in this study could be due to inability to secrete ExoU *in vitro*, despite harboring the *exoU* gene. It has been shown that a small 15-kDa chaperone protein specific Pseudomonas chaperone for ExoU (SpcU), encoded by *spcU* downstream of *exoU*, forms a complex with ExoU and maintains the N-terminus of ExoU in an unfolded state, which is required for ExoU secretion (38, 46). Indeed, our WGS data showed that the non-virulent strain PA AP contained the gene *spcU*, but there was a point mutation mutation of T→G at nucleotide position 280, causing phenylalanine (Phe) to be exchanged by valine (Val) at amino acid (aa) 94 of SpcU (SpcU-F94V) (Fig. 6), which may account for its loss of cytotoxicity in this strain.

### Evolutionary characteristics of the *exoU+* genotype

**Fig 7:**
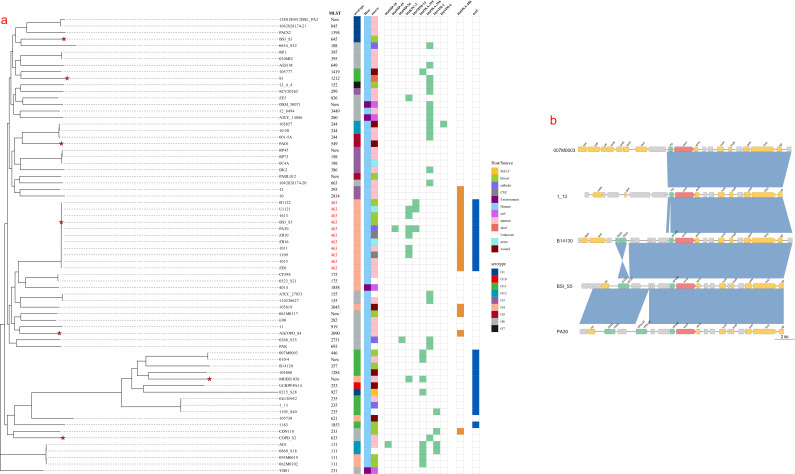
The ST463/O4 clone harboring the *exoU* and multidrug resistance genes and the genome organization of *exoU* loci with insertion sequence IS3 families. Full-genome single-nucleotide polymorphism-based phylogenetic and characteristics of all isolates. Strains sequenced in this study are marked with asterisk (**a**). IS families adjacent to *exoU* in BSI_S5 genome as defined in Prokka (**b**).

The confirmation of the role of *exoU* in virulence and prevalence prompted us to examine *P. aeruginosa* clinical isolates for its evolutionary characteristics. We selected 63 strains with known genome sequences combined with our sequenced seven strains to construct single-nucleotide polymorphism-typing phylogenetic tree analysis (Fig. 7a), to investigate the potential origin and the history of the spread of *P. aeruginosa* strains containing the *exoU+* genotype. Phylogenetic tree analysis showed that clinical isolates from various sources do not cluster closely. MLST and serotype analysis revealed that the 70 isolates belonged to 49 types and 9 serotypes, demonstrating the sequence diversity of *P. aeruginosa* clones was high among different sources. The highly virulent strain BSI_S5 belonged to ST463/O4, which is consistent with a previous observation, where ST463/O4 isolate was found to be a potential high-risk clone (47). Remarkably, carbapenem resistance (CR; mainly *blaKPC–2*, *blaOXA-396*, and *blaOXA-395*) was identified in 51.43% (36 of 70) of the isolates and 63.64% (7 of 11) of ST463/O4. Among the ST463/O4, the other four strains would also be resistant to β-lactams because of the presence of intrinsic resistance genes *blaOXA–486*, along with the aminoglycoside-resistance gene (*aph(3′)-IIb*) and fluoroquinolone-resistance gene (*crpP*). Indeed, MIC determination confirmed that BSI_S5 was highly resistant to fluoroquinolone ciprofloxacin and β-lactams cefoperazone, aztreonam, piperacillin, ceftazidime, cefepime, piperacillin/tazobactam, imipenem, and meropenem, but was susceptible to amikacin and tobramycin (Table S2). Thus, the ST463/O4 strain was coexisting with multidrug resistance genes, especially CR.

To further explore whether there is any relationship between ST types and presence of *exoU* in *P. aeruginosa*, we found that ST463, ST446, ST357, ST1284, ST253, ST927, ST235, ST621, and ST1853 displayed the *exoU*+ genotype (22 strains in total); while notably, 11 *exoU*+ isolates were ST463/O4 and 3 were ST235/O11. The *P. aeruginosa* ST463 and ST235 serotypes are often associated with poor clinical outcomes due to its multidrug resistance and virulence factors (19, 48, 49).

### Mobile genetic elements in *P. aeruginosa* BSI_S5

Like many bacterial pathogens, *P. aeruginosa* genome is highly plastic and complex, composed of the core genome interspersed with DNA segments constituting mobile genetic elements (MGEs, a variety of accessory genes that form part of the pan-genome). The MGEs embrace plasmids, bacteriophages, genomic islands, integrative and conjugative elements, integrative and mobilizable elements, insertion sequences (ISs), etc., which are considered as major contributors to the spread of antimicrobial resistance genes and virulence factors via horizontal gene transfer. To facilitate the understanding of specific regulatory mechanisms of *exoU* in BSI_S5, we analyzed MGEs present in its genome adjacent to *exoU*. As shown in Fig. 7b, there are two types of ISs present, IS222 and ISPa32, which belong to the IS3 family. Notably, *exoU* is located downstream of ISPa32. ISPa32 was originally detected in *P. aeruginosa*, with 80% (ORFA) and 95% (ORFB) amino acid similarity to IS407. Its complete sequences had three open reading frames (ORFs), and the third ORF is a putative ORFAB transposase (50). Further alignments showed that the IS sequences adjacent to *exoU* in BSI_S5 are quite similar to those of PA30, B14130, except some inversion or insertions of IS before the ISPa32 (Fig. 7b). Activation of neighboring gene expression can occur in two principal ways: either via promoters contained entirely within the IS driving transcripts that escape into neighboring DNA or by the formation of hybrid promoters following insertion (51). IS activity can result in increased resistance to antibiotics or insertional inactivation of specific porins (51, 52). Besides, ISs are known to promote genome reshaping and may even affect the bacterial dependence on its host (51). ISs of *P. aeruginosa*, mainly those of the IS3 family, have been shown to be related to genome rearrangements (53). In the genome of BSI_S5, although two types of ISs (from the IS3 family) adjacent to *exoU* were observed, there is no clear evidence showing these ISs are implied in large-scale rearrangements. Nevertheless, the current analysis showed that ISPa32 is a species-specific sequence that may have the potential to transfer its neighboring gene (i.e., *exoU*) to other strains via horizontal gene transfer. Further exploration of the relationship between ISPa32 expression and transposition activity is essential to understanding the dynamics of ISs in *exoU* expression and transfer in *P. aeruginosa*.

## DISCUSSION

In this study, we used the comparative genomic analysis followed by gene knockout and complementation to investigate the unique genes affecting the pathogenicity of *P. aeruginosa*. We found that *exoU*, *chr_1696*, and *chr_4238*, which encode the T3SS effector-ExoU, T1SS permease/ATPase or polysaccharide biosynthesis protein, respectively, are specifically present in the highly virulent *P. aeruginosa* BSI_S5 strain, which belongs to ST463/O4. Deletion of *exoU* showed significantly attenuated cytotoxicity, while deletion of the two other unique genes caused no apparent loss of cytotoxicity compared to the parent strain BSI_S5; and complementation of *ΔexoU* mutant restored its cytotoxicity.

To verify the role of ExoU during infection, *P. aeruginosa* BSI_S5 (WT) and *ΔexoU* were employed in a bloodstream infection utilizing a mouse model via tail vein. The increased survival rate of mice infected with *ΔexoU* compared to WT showed that *ΔexoU* was significantly attenuated for virulence *in vivo*, and the complementation of the *ΔexoU* mutant completely restored its virulence. Bacterial recovery showed that a homogeneous distribution of the *P. aeruginosa* strain in the liver when inoculated with low dose of parent strain or *ΔexoU,* which indicated that deletion of *exoU* gene in the strain did not affect its liver survival but only changed its survival in the lung and spleen. No *P. aeruginosa* spp. were detected in the GBs and feces whether infected by low dose of parent strain or *ΔexoU.* With high dose of *P. aeruginosa*, there was a significant difference in the bacterial loads between the two groups (spleen and feces), and the bacterial population in the GBs was dramatically expanded in the majority of mice infected by parent strain while not in the *ΔexoU*-infected mice. Regardless of low or high dose used, there was also a significant difference in bacterial population in the lung or spleen, while the bacterial numbers in GBs or feces differed significantly between parent strain and *ΔexoU*-infected mice. It could be speculated that during bacteremia, a small subpopulation of *P. aeruginosa* replicated in the liver and seeded the GB. Once in the GB, this population then expanded dramatically, disseminated to the intestines, and was excreted in the feces. Following the liver–GB–intestinal excretion pathway, *P. aeruginosa* from systemic infections could return to the environment in high numbers in order to enhance transmission between mice (34). Moreover, the hematoxylin and eosin (H&E) staining showed that ExoU induced severe lung lesions and the mucosal damage to GB. Taken together, these results demonstrated that *ΔexoU* was attenuated *in vivo* compared with the parent *P. aeruginosa* strain, underscoring it as a strong candidate for further targeting to improve treatment of *P. aeruginosa* systemic infections. This supports that T3SS is a major driver of virulence in *P. aeruginosa* and is known to damage epithelial surfaces through direct injection of cytotoxic effector proteins. Moreover, ExoU can significantly contribute to the colonization of *P. aeruginosa* in the lung, liver, spleen, GB, and feces of the host with acute bloodstream infection.

We also found that a strain PA AP harbored the *exoU* gene but showed low cytotoxicity. This indicates the presence of *exoU* gene by PCR detection alone is not sufficient for identifying ExoU expression strains for virulence testing. Instead, an intact protein SpcU encoded by *spcU* downstream of the *exoU* is crucial for ExoU secretion and virulence expression, as we identified a clinical isolate with intact *exoU* gene but had a point mutation T→G at nucleotide position 280 (mutation from Phe to Val at amino acid 94) in *spcU* that caused loss of cytotoxicity. Since strains harboring and expressing *exoU* may cause particularly severe disease in patients, it would be of interest to develop a rapid molecular test to detect the high virulence phenotype by detecting not only the presence of the *exoU* gene but also intact downstream *spcU* by targeted sequencing of both genes. It is worth noting that although SpcU was previously found to be essential for ExoU secretion by sequential deletion of the gene loci using lab experimental system (38), this study is the first to identify a point mutation in SpcU in a clinical isolate responsible for the loss of its function in causing defective cytotoxicity. Although our results clearly show that the intact coexistence of *spcU* and *exoU* are required for exerting toxicity of *P. aeruginosa*, one limitation in this study is that we could not elucidate how the amino acid change affects the functional domains in SpcU that interrupts the secretion of ExoU. Further experimental work will be necessary to elucidate the nature of the ExoU and SpcU interaction through structural biology studies.

In addition, our results indicate that clinical isolates from various sources do not cluster closely and belong to different ST types. Notably, the highly virulent strain BSI_S5 is serotype ST463/O4, which is an emerging high-risk clone that spread rapidly in East China, whose genome commonly contains *exoU* and multidrug resistance genes, especially CR. The *exoU* gene is present in all of the ST463/O4 genomes but not specific for this lineage. The combined extensive drug resistance and the high virulence due to *exoU* may contribute to its high pathogenicity and mortality in the host and spread in hospital environments. Besides, the two types of ISs (IS222 and ISPa32 belonging to the IS3 family) adjacent to *exoU* may potentially influence *exoU* transfer to different strains. Finally, the exact mechanism by which ExoU induces cellular death and its role in pathogenesis of *P. aeruginosa* ST463/O4 will be further investigated in the future.

In conclusion, through a series of whole-genome sequencing, molecular biology and cellular and animal studies, we found that the type III secretion system effector-ExoU was the main determinant of pathogenicity of the highly virulent *P. aeruginosa* clinical isolate BSI_S5, which caused serious systemic infections and belonged to serotype ST463/O4. We showed that deletion of *exoU* but not two other unique genes, *chr_1696* and *chr_4238*, caused significant attenuation of cytotoxicity and virulence, while complementation of the *ΔexoU* mutant restored cytotoxicity. Interestingly, we found that a clinical isolate with intact *exoU* gene but harboring a mutation of T→G substitution at nucleotide position 280, causing amino acid Phe to Val change at aa 94 of SpcU (SpcU-F94V), showed significant loss of cytotoxicity, suggesting that a functional downstream SpcU is required for ExoU secretion and cytotoxicity. In addition, we assessed a total of 77 (5 + 72) clinical isolates of *P. aeruginosa* and found that *exoU*+ genotype comprised 11.68% (9 of 77) of these strains (excluding 63 strains with known genome sequences and PAO1). Our study demonstrates the important role of not only ExoU but also its downstream intact SpcU in conferring virulence of *P. aeruginosa* and calls for surveillance of both *exoU* and *spcU* by targeted sequencing for improved detection and control of virulent *P. aeruginosa* infections in the clinical setting.

## MATERIALS AND METHODS

### Bacterial strains

Human sputum, blood, or stool samples from patients diagnosed with bloodstream or lung infections were obtained from an already existing collection of The First Affiliated Hospital, Zhejiang University School of Medicine, in 2020. The identity of *P. aeruginosa* was confirmed by *Pseudomonas* isolation agar (PIA) plates and 16S ribosomal DNA sequencing.

### Whole-genome sequencing

Whole-genome sequencing of clinical isolates of *P. aeruginosa* and the common model strain *P. aeruginosa* PAO1 included as a control strain was conducted by Personalbio (Jiangsu, China). Briefly, the WGS was performed using Illumina NovaSeq sequencing platform and also PacBio Sequel sequencing platform in order to obtain accurate and complete whole genome sequences. A total of two libraries were constructed for each strain for the WGS sequencing.

### Measurement of virulence using chicken embryo infection model

Chick embryo infection model was used to evaluate the virulence of *P. aeruginosa* strains. Hatching eggs were obtained from a hennery in Deqing, Zhejiang Province. A batch of 10-day-old chick embryos were used in this study. A hole (1.2-mm diameter) was aseptically drilled in the egg shell using a needle, and a suspension (100 µL, 10^5^ CFU/egg) of *P. aeruginosa* strains being tested was inoculated intra-allantoically, followed by aseptic sealing of the inoculation hole with sterile medical pressure-sensitive adhesive tape. Two controls were included, including eggs inoculated with PBS (100 µL) and eggs not subject to any inoculation treatment. Each run contained 10 eggs for each bacterial strain and each control as described (21). The eggs were checked by ovoscope and candle at different time intervals to evaluate the viability of the embryos. Dead embryos were identified by the absence of spontaneous movement (usually associated with hemorrhage), loss of vascular architecture, and abnormal morphology. Such eggs were removed from the incubator, and the stage of development was assessed using Hamburger and Hamilton’s key. Incubation was terminated on the 17th day of embryogenesis, when all live embryos were euthanized.

### Cytotoxicity assay

The cytotoxicity assay was performed as described with some modifications (54). Briefly, six-well tissue culture plates were seeded with 5 × 10^5^ THP-1 cells/mL cultured in RPMI 1640 medium (supplemented with 10% fetal bovine serum (FBS), 100-U/mL penicillin and streptomycin). Cells were maintained at a density of 1 × 10^5^–10^6^/mL and differentiated by addition of 100-ng/mL phorbol 12-myristate 13-acetate 24–48 hours prior to infection. The cells were washed two times with PBS to remove non-adherent cells and fresh RPMI 1640 medium (supplemented with 10% FBS) was added (for lactate dehydrogenase assay, fresh RPMI 1640 medium was supplemented with 1% heat-inactivated FBS). Cells were incubated at 37°C under humidified atmosphere and 5% CO_2_ at least 30 min prior to infection with *P. aeruginosa*. THP-1 cell cytotoxicity was determined by incubation of cells with overnight cultures of *P. aeruginosa* at a multiplicity of infection (MOI) of 10:1 in 2.0 mL of assay medium. The infected cells were incubated for 3.5 hours at 37°C and 5% CO_2_ before cell viability (trypan blue stain) was evaluated. Supernatants were harvested and analyzed for LDH activity using an LDH Activity Assay Kit (Beyotime Biotechnology, Shanghai, China) according to the manufacturer’s instructions.

### Flow cytometry analysis

THP-1 cells (5 × 10^5^ cells/mL) were infected with *P. aeruginosa* for different times (MOI = 10:1). Cells in medium alone were set as the control. Apoptotic cells were investigated by double staining with annexin V/PI using an annexin V-EGFP apoptosis detection kit (Cellor Lab, Shanghai Epizyme Biomedical Technology Co., Ltd) according to the manufacturer’s instructions. Cells were centrifuged at 200 × *g* for 3 min, and washed twice with ice-cold PBS (pH 7.4). The cell pellet was resuspended in 200 µL of binding buffer. Cells were stained by adding 4 µL each of FITC-annexin V and PI working solutions. After incubation at room temperature for 10 min in the dark, samples were analyzed by flow cytometry (CytoFLEX, Beckman) within 1 hour.

### Knockout mutant construction in *P. aeruginosa*

All bacterial strains were grown on LB agar or LB broth prior to experimentation. For plasmid preparation, *E. coli* transformants were cultured in terrific broth (12-g/L peptone, 24-g/L yeast extract, 9.4-g/L dipotassium hydrogen phosphate, 2.2-g/L potassium dihydrogen phosphate, and 4-mL/L glycerol). PIA (20-g/L peptone, 1.4-g/L MgCl_2_, 10-g/L K_2_SO_4_, 25-mg/L Irgasan, and 13.6-g/L agar) supplemented with 4% glycerol was used in the selection of the first homologous recombination event. Tryptone yeast extract and sucrose (TYS10) (10-g/L tryptone, 5-g/L yeast extract, 10% sucrose, and 15-g/L agar) plates were used in the selection of the second homologous recombination event. Antibiotics were added to *P. aeruginosa* cultures, where appropriate, at the following concentrations in microgram per milliliter: kanamycin, 2,000; gentamicin, 50–150; or carbenicillin, 300–500. *E. coli* cultures were supplemented with antibiotics at the following concentrations in microgram per milliliter: kanamycin, 50; gentamicin, 10; or ampicillin, 50.

Partially overlapping flanking primers were designed to amplify the upstream and the downstream 5,000-bp DNA sequences of *chr_1696*, *exoU*, and *chr_4238* of *P. aeruginosa* (Table S1). The upstream and downstream of the gene of interest were amplified by PCR as follows: 10 ng of upstream primer and downstream primer, 100 ng of template DNA, 25 µL of 2× Phanta Flash Master Mix (Vazyme, Nanjing, China), added to ddH_2_O to a final volume of 50 µL. The thermo cycling parameters were programmed according to the following protocol: 98°C for 30 s, then 30 cycles at 98°C for 10 s, Tm for 5 s, and 72°C for 15 s, then a final extension at 72°C for 1 min.

pK18mob-sacB plasmid was propagated in *E. coli* using LB medium containing 50-µg/mL kanamycin and incubated at 37°C for 16 hours. The cultures were collected and the plasmid DNA was purified using TIANprep Mini Plasmid Kit (TIANGEN, Beijing, China) according to the manufacturer’s instructions. To generate the linearized overlapping fragments of pK18mob-sacB, DNA was digested with *SaI*I (Thermo Fisher). The PCR products and the linearized vector were analyzed by DNA agarose gel electrophoresis and purified by gel extraction kit (TIANGEN) and dissolved in TE buffer, then quantified by Nanodrop (Thermo Fisher). The upstream and downstream of each gene were inserted into pK18mob-sacB using ClonExpress II One Step Cloning Kit (Vazyme) to obtain *pK∆1696, pK∆exoU,* and *pK∆4238,* respectively. The newly assembled products were transformed into *E. coli* DH5α competent cells (Vazyme) via heat shock. Subsequently, triparental matings were used to mobilize plasmids from *E. coli* DH5α to *P. aeruginosa* with the conjugative helper strain *E. coli* HB101 (pRK2013) (55). The cells were plated on PIA plates containing 2,000-μg/mL kanamycin (PIA/Kan 2,000), and incubated at 37°C overnight. Colonies growing on the PIA plates were checked by colony PCR using the pK18mob-sacB forward primer and a primer specific to the genomic DNA adjacent to the cloned 500-bp downstream sequence (Table S1). Positive colonies were purified by streaking on new (PIA/Kan 2,000) plates. Purified merodiploids were streaked on TYS10 plates and incubated at room temperature (~20°C) for 72–96 hours. Colonies were examined by colony PCR with primers specific to the genomic DNA before the cloned 500-bp upstream sequence and after the cloned 500-bp downstream sequence of each deleted locus (Fig. S1). Colonies with the confirmed deletion were purified by streaking on a TYS10 plate and further verified by Sanger sequencing (Tsingke Biotech Co., Ltd.; Fig. S4-1 through S4-3).

### Gene complementation

For gene complementation, the locus of *exoU* as well as its upstream and downstream was amplified using appropriate primers (Table S4), purified, and seamlessly fused with the linearized pK18mob-sacB. The resulting plasmid was transformed into *E. coli* DH5α. Colonies containing the complement construct were confirmed by colony PCR using the universal M13 primer pair. Positive colonies were further selected for DNA preparation and sequencing. The fused plasmid was then transformed into the *ΔexoU* mutant by electroporation followed by two rounds of screening (the same procedure as above). Finally, two populations were identified on the TYS10 plates: one included the desired complementation strain [*ΔexoU* (*exoU*)], while the other included wild type revertants (*ΔexoU)*. As a result, the complementary strain *ΔexoU (exoU)* carries a 2,064-bp DNA fragment *exoU*, and the sequences were verified by DNA sequencing (Fig. S4-4).

### Polymerase chain reaction (PCR) amplification of the *exoU* gene

The prevalence of the *exoU* gene in different clinical isolates was determined by PCR. Briefly, *exoU* was amplified with primers (*exo*U-F: ATGCATATCCAATCGTTGGG, *exoU*-R: TCATGTGAACTCCTTATTCCGC). PCR was carried out as follows: 2-µL template genomic DNA (50–100 ng), 10 µM of each primer, 12.5 µL of 2 × Phanta Flash Master Mix (Vazyme), added ddH_2_O to a final volume of 25 µL. The DNA was amplified using the following protocol: 98°C for 30 s, then 30 cycles at 98°C for 10 s, Tm for 5 s and 72°C for 15 s, then a final extension at 72°C for 1 min. PCR products were separated in 1% agarose gel for 30 min at 120 V, stained with Gel-Green (Beyotime, China) and detected by iBright CL750 Imaging System (Thermo Fisher Scientific).

### Mice

Seven- to nine-week-old female BALB/c mice were purchased from Hangzhou Medical College and housed in a specific-pathogen-free facility at Zhejiang Academy of Medical Sciences.

### Mouse model of tail vein injection

Overnight cultures of *P. aeruginosa* were grown in 5  mL of LB medium and resuspended in PBS prior to infection. The tail veins of 7- to 9-week-old BALB/c female mice restrained using a GEGD-Q Tailveiner [Globalebio (Beijing) Technology Co., Ltd, China] were dilated with a heat lamp. A defined number of *P. aeruginosa* in 50  µL of PBS was injected into the tail vein of mice using a 29-gauge needle. Inocula were confirmed by plating serial dilutions on LB agar. At specified times post-infection, the mice were anesthetized and sacrificed by cervical dislocation. To monitor bacterial loads, organs were excised, weighed, and homogenized in PBS. Viable bacteria were enumerated by plating serial dilutions of either fecal pellets or organ tissue homogenates on PIA agar. Recovered colonies of bacteria were counted to determine total CFU per gram organ.

For histological sections, 7- to 9-week-old female BALB/c mice were infected via tail vein. At defined points post-injection, GBs and lungs were harvested and fixed in 4% paraformaldehyde solution for a minimum of 48 hours. Samples were then paraffin embedded, processed, stained with H&E, and mounted on glass slides. For light microscopy analysis, histological images were obtained using a digital camera (BX53, Olympus).

## SUPPLEMENTAL MATERIAL

Fig. S1 to S4, Tables S1 to S5 spectrum.02224-23-s0001.docx Supplementary data.

ASM does not own the copyrights to Supplemental Material that may be linked to, or accessed through, an article. The authors have granted ASM a non-exclusive, world-wide license to publish the Supplemental Material files. Please contact the corresponding author directly for reuse.
