# Supplementary material for: The type III secretion system facilitates systemic infections of Pseudomonas aeruginosa in the clinic
Source: Microbiol Spectr. 2023 Dec 13;12(1):e02224-23. doi: 10.1128/spectrum.02224-23 (PMC10783026; doi:10.1128/spectrum.02224-23)
Supplement: Fig. S1 to S4, Tables S1 to S5 — Supplementary data. [file spectrum.02224-23-s0001.docx]

**Supplementary Information**

**Table S1** Information of clinical background and different *P. aeruginosa* clinical isolates investigated in this study.

| **Isolates** | **Abbreviation** | **Sex** | **Age** | **Isolation year** | **Sample source** | **Primary diagnosis** |
| --- | --- | --- | --- | --- | --- | --- |
| S1 | S1 | Male | 49 | 2021 | Stool | CH, hydronephrosis (right) |
| COPD_S2 | S2 | Male | 67 | 2021 | BALF | COPD |
| BSI_S3 | S3 | Male | 78 | 2021 | Blood | AML |
| AECOPD_S4 | S4 | Male | 87 | 2021 | Sputum | PE, COPD with acute exacerbation |
| BSI_S5 | S5 | Female | 53 | 2021 | Blood | AML |

BALF: bronchoalveolar lavage fluid, CH: cerebral hemorrhage, AML: acute myeloid leukemia, COPD: chronic obstructive pulmonary disease, PE: pulmonary embolism.

**Table S2** Minimum inhibitory concentrations (MICs) of some antibiotics for the six strains of *P. aeruginosa*

| **Category** | **Strains Antibiotics** | **PAO1** | **S5** | **S1** | **S2** | **S3** | **S4** |
| --- | --- | --- | --- | --- | --- | --- | --- |
| Carbapenems | MEM | 0.5 | R | 128 (R) | 0.5 | 8 | ≤ 0.25 |
|  | IMP | 1 | 64 (R) | 32 | 0.5 | 8 | 0.5 |
| Cephalosporins | CAZ | 0.5 | 32 (R) | 64 (R) | 8 | 2 | 0.5 |
|  | FEP | 0.5 | R | R | 1 | 2 | 1 |
|  | CFP | 1 | R | R | 8 (I) | 8 (I) | 2 |
| Monobactams | AZT | 2 | R | R | 8 | 8 | 2 |
| Cephalosporins / Penicillins + β-Lactamase inhibitor | CAZ/AVI | NT | R | NT | NT | NT | NT |
|  | T/C | NT | R | NT | NT | NT | NT |
| Fluoroquinolones | CIP | 0.125 | 32 (R) | 4 (R) | ≤ 0.125 | ≤ 0.125 | ≤ 0.125 |
|  | CLI | 0.125 | 4 | 0.5 | 0.25 | ≤ 0.125 | ≤ 0.125 |
| Aminoglycosides | TOB | 0.5 | 1 | 0.5 | 0.5 | 0.5 | 0.5 |
|  | AMK | 2 | 8 | 2 | 2 | 2 | 2 |
| Polymyxins | CST | 1 | 2 | 1 | R | 1 | 1 |

MEM: meropenem, IMP: imipenem, FEP: cefepime, CFP: cefoperazone, AZT: aztreonam, CAZ: ceftazidime, AVI: avibactam, T/C: ticarcillin/clavulanate acid, CIP: ciprofloxacin, CLI: clinafloxacin, AMK: amikacin, TOB: tobramycin, CST: colistin.

Breakpoints for resistance (μg/ml) according to CLSI (2018b) : MEM≥8, IMP≥8, CAZ≥32, FEP≥32, FEP≥16, AZT≥32, CAZ/AVI≥16/4, T/C≥128/2, CIP≥4, TOB≥16, AMK≥64, and CST≥8.

NT: Not tested, R: resistant, I: intermediate, ‘R’ without value means the MIC ≥ 128 μg/ml, Value without any symbol means ‘susceptible (S)’.


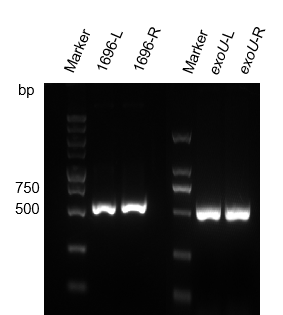
 **
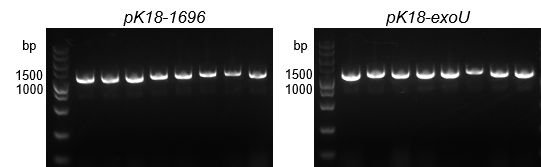
**

(a) (b)

*
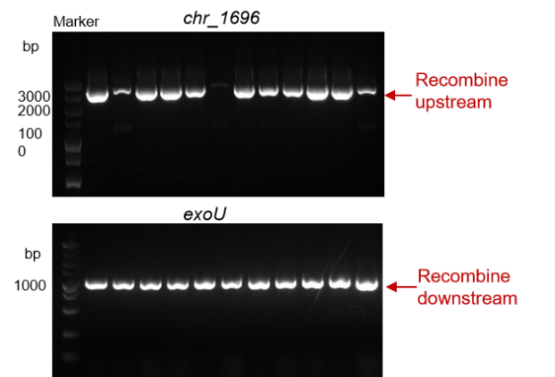

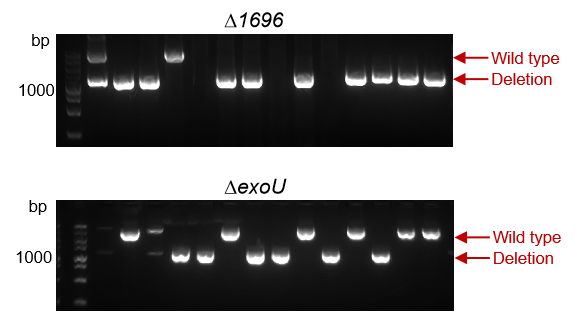
*

(c) (d)

**Figure S1**. **Supplemental data corresponding to gene knockout study.** Amplification of the 500 bp upstream and downstream DNA fragments of 1696 and *exoU*, respectively. L, the upstream genomic DNA sequence of each gene; R, the downstream genomic DNA sequence of each gene (a); Colony PCR of fused deletion alleles. 10 μl of PCR products of several colonies randomly selected, all of which showed the 1000 bp fused deletion alleles (b); Colony PCR of merodiploids. 10 μl of PCR product of a colony in which plasmid integrated via the downstream homologous sequence (*exoU*); 10 μl of PCR product of a colony in which plasmid integrated via the upstream homologous sequence (*chr_1696*) (c); Screen of deletion mutants by colony PCR. 10 μl of PCR products of 14 colonies randomly selected from the TYS10 plate. For ∆*1696,* fourteen colonies were randomly selected, 8 colonies were deletion mutants while six colonies reverted to wild type during the second recombination event (d).

**Table S3** Primers used in this study for gene knockout construction

| **Primer name** | **Primer sequence** |
| --- | --- |
| *exoU*-LF | cggggatcctctagagtcgacGCTGGACGAGATGGCGCG |
| *exoU* -LR | ccatgtatcaaGTTCGCTCCTTGAAATACCGAG |
| *exoU* -RF | ggagcgaacTTGATACATGGCTGGCACAGTG |
| *exoU* -RR | cttgcatgcctgcaggtcgacTGAACAGATAGGCCACCTTCG |
| YZ-*exoU-F* | ATCAGCGGTTTTCGTGTCAC |
| YZ-*exoU-R* | GAAATGGTGTTGGCACGACA |
| *1696*-LF | cggggatcctctagagtcgacCTCGATTCAAGCGCGCCA |
| *1696*-LR | ccatgtTGCATTGCTTCCGTGCTGG |
| *1696*-RF | acggaagcaatgcaACATGGGCGCGCAAAGAG |
| *1696*-RR | cttgcatgcctgcaggtcgacAGCCAGGGAGGTTGGAGCG |
| YZ-*1696*-F | CGGAAGTCCTCGTTGCTGA |
| YZ-*1696*-R | TAGCCTGGGGAATCAACGG |
| *4238*-LF | cggggatcctctagagtcgacTGCCAGACTTATGTCTGGAGTCTG |
| *4238*-LR | ccctcatgccATTAAACCTCCTGCAGTGAATCATAT |
| *4238-*RR | gaggtttaatGGCATGAGGGTCTTAATAACCG |
| *4238-*RF | cttgcatgcctgcaggtcgacGCCGTAGGATCATTTATCTGAATG |
| YZ-*4238-*F | GGAGTCTGTGGTCAACTGGG |
| YZ-*4238*-R | ACATCATCGATATAAACGAGCGT |
| pK18mob-sacB-F | TACCCGGGGATCCTCTAGAG |
| pK18mob-sacB-R | CTTGCATGCCTGCAGGT |
| M13 Forward | GTAAAACGACGGCCAG |
| M13 Reverse | CAGGAAACAGCTATGAC |

**Table S4** Primers used in this study for gene complementation

| **Primer name** | **Primer sequence** |
| --- | --- |
| *∆exoU (exoU)*-LF | cggggatcctctagagtcgacGTCGACATCTTGGTGGAGTACGG |
| *∆exoU (exoU)*-LR | tggatatgcatGTTCGCTCCTTGAAATACCGAGG |
| *∆exoU (exoU)* -eL | ggagcgaacATGCATATCCAATCGTTGGGG |
| *∆exoU (exoU)* -eR | gtgccagccatgtatcaaTCATGTGAACTCCTTATTCCGCC |
| *∆exoU (exoU)*-RF | gaTTGATACATGGCTGGCACAGTG |
| *∆exoU (exoU)*-RF | cttgcatgcctgcaggtcgacGATGGGGCTCGGTAGAAATGG |

**Table S5** Strains and vectors used in this study

| **Strains or plasmid** | **Characteristics** | **Source or reference** |
| --- | --- | --- |
| **Strains** | | |
| *Pseudomonas aeruginosa* BSI_S5 | Wild-type | Clinical strain |
| *∆4238* | BSI-S5 *chr_4238* deletion mutant strain | This work |
| *∆1696* | BSI-S5 *chr_1696* deletion mutant strain | This work |
| *∆exoU* | BSI-S5 *exoU* deletion mutant strain | This work |
| *∆exoU (exoU)* | BSI-S5 *exoU* complementation mutant strain | This work |
| **Plasmid** | | |
| pK18mob-sacB | Kan^R^, mob+, *lacZa*; used for gene integration into the host chromosome | Simon R, et al. 1983 |
| pRK2013 | Kan^R^, mobilization helper plasmid | Knauf VC, Nester EW., 1982 |
| *pK∆1696* | Kan^R^, pK18mob-sacB derivative, carries a 2148 bp deletion of *chr_1696* | This work |
| *pK∆exoU* | Kan^R^, pK18mob-sacB derivative, carries a 2064 bp deletion of *exoU* | This work |
| *pK∆4238* | Kan^R^, pK18mob-sacB derivative, carries a 1035 bp deletion of *chr_4238* | This work |
| *pK∆exoU (exoU)* | Kan^R^, pK18mob-sacB derivative, carries a 500 bp upstream, 500 bp downstream and 2064 bp complementation of *exoU* | This work |

**Figure S2**. **Growth curves of deletion mutants and their complementation strains.** Overnight cultures were diluted at 1:100 in fresh LB and cultured at 37 ^o^C with shaking and the optical density at 600 nm was monitored by BioTek spectrophotometer. Three biological replicates were performed; error bars represent the standard error of the mean of the biological replicates.

**a**

**b**

**
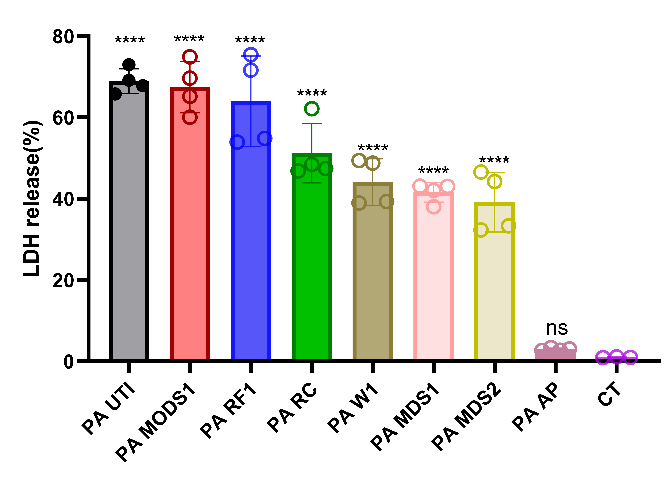
**

**Figure S3**. **Cytotoxicity of candidate gene mutations and different strains.** (**a**) Deletion of *exoU* but not genes 1696 or 4238 caused decreased cytotoxicity for THP-1 macrophages. LDH assay at 4.5 h.p.i. CT: control, uninfected; WT: wild type (*P. aeruginosa* BSI_S5). (**b**) A strain PA AP harboring the *exoU* gene but showed low cytotoxicity. Cell viability was determined by LDH assay, MOI=10:1, 2 h.p.i. Data are presented as Mean ± SD of at least three biological replicates. ^ns^*p* > 0.05, **** *p* < 0.0001 (one way ANOVA) as compared to CT (un-infected cells).


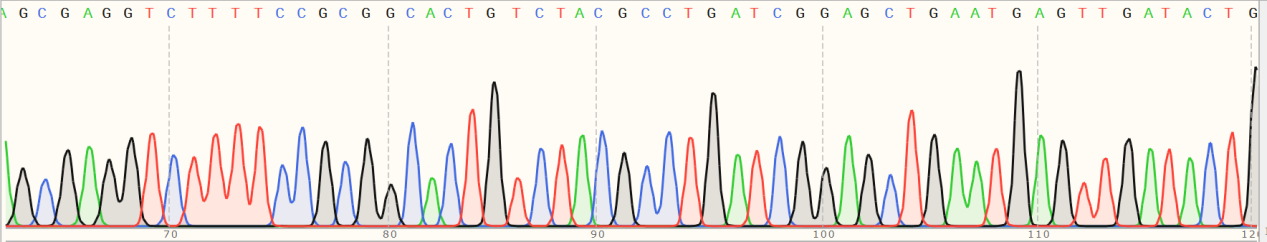


>*∆1696*

GGGGCTTCTCACGGCGTCGAGGCGCTCGTCGACTCGATTCAAGCGCGCCAGGATCGCCTCTAGCGAGGTCTTTTCCGCGGCACTGTCTACGCCTGATCGGAGCTGAATGAGTTGATACTGCTGGTAGCCCAGCAGGCCGGCCATTGCTGTCAAGCAGAGGCCAAGAATCATTGATGCAGGGGATGGGCGTTTCATGAGGGTCCTCGCGCGAAGCCAGAGACACCCTCTCAGACTGCCCCCATTGAGGCAGTAGGAAATACTCGGTGAGTGGGGGTGGGATTTTTTTGGATAGGATTCTGGCGAATCGCCGGGCCTGATGCGCTATGAGAGGATGACGCGACTCTGGGGGCCTTTCGCCGCTTGATCTATGCGATTAAGTTTGGTCGAGCCGGTAACCGGTGTCCTGTGTTTCAAGGGGCTCGTTCAGATACCAATCCAAGCATTCATACTAAGAGTGTGATTCTGTTCGTGTCATCTTTGGTGCTAAAGCTGCTATCTAGAGAGTTCCTTACCCCAGCACGGAAGCAATGCAACATGGGCGCGCAAAGAGAACTGCTGCAGCGCTATCGGCGTGCCTGGTCGCACTCGTGGCACAACCGAACAAAACTGGACACACCGCTGCGCAGCCAGCACGAAGTGCATTTCCTCCCTGCGGCACTGGCGCTGCAGGAGCAGCCAGTGCATCCCGCCCCGCGCTATATCCAGTGGGCGATCATGCTTTTCGCGGCGCTGGCCTTGGTCTGGGCGTGCATCGGTAAGATCGAGGTGGTTGCCACGGCAAGCGGCAAGGTCGTCCCGAGCGGTAGGAGCAAGGTCATCCAGCCCAGCGATGTCGCCGTAGTCAAATCGATCCATGTCCGCGATGGGCAGTTGGTGAGAGCGGGCGAATTGCTGGTCGAACTGGACTCCAATATCACCGGCGCGGACGTCGACCGGCTGAAGAGTGATCTGCTCGCTGCCCACATCGATAGCGCCCGAGCCGCTGCGTTGCTTGACGCCTTCGACACCTAGCGTCCACCCCTGCGCGG

**Figure S4-1**. DNA sequencing to confirm deletion mutant *∆1696* (up/down streams of deleted genes).


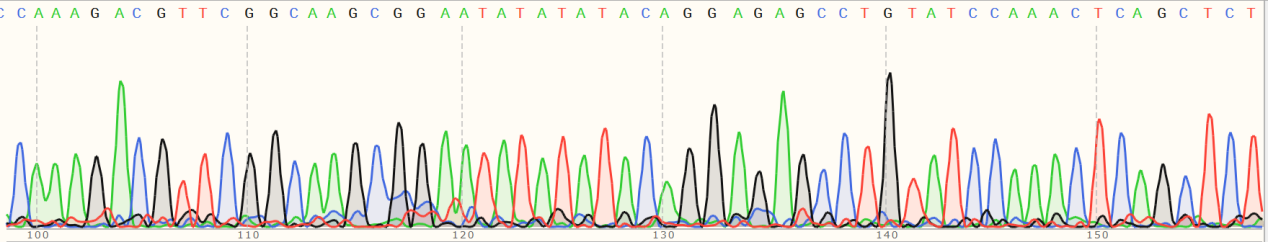


>*∆4238*

ATTTATCCGAAGGGACATTTATCTCCATGGGATAACCCTGGCAATATTATAGCAAAAAGTCGCCACCGCAGAGTTATAACTGGGACGCGACCACTTGCCAAAGACGTTCGGCAAGCGGAATATATATACAGGAGAGCCTGTATCCAAACTCAGCTCTCGAATATAATTCTCTGCATCGCGCTTACTCAGCCCATATTTATTCTGCAACTCCGCCTGCGTCGAAGATGTATAGAGCAGAGGAATAGAACGACCAGTTCGCCGGATTGCATCACATAGTGCTTTGGTCAGATTCGCATTTCCAGATTTGAAGTCCTCAGGATTCTCGGGGCGATTGACACCAGCCAAATGAAAAACAAAATCAATCGAGCCAAGAATTCCTGGCAAGCTTTCAATACTATTCTCGCGCGTAAAGGTTATAACCTCGACATCATTTCGCTCAGCTAAATGAGCAGCAAGGTTCTTTCCTACAAAGCCATTTGCACCGGTTATTAAGACCCTCATGCCCTATTCCTCGGGGTTTGCCTGTTCGCCACGCTGAATAGCACGTATGAAATCAAGCTTCAGCAGCAATTCCTGCATACCCTTGACATCCAGACGATCTGTATTGTGTGAATTATAGTCTTCGGTATGGGAAATCCTTTCCTCACCTTGCTCTACAAACTTACTATAATTCAGATCGCGCAAATCCGGCGGTACGCGATAATAGTCTCCCATATCTTCGGCACAAGCCATTTCCTCACGACTCAAGAGCGCTTCATAGAGTTTCTCGCCATGCCCGCGTACCGATTACATTGACCGGATAGCCTTCTTTACCAAGCAGCCCCGTCAATGCCGTGGGCCAGCACCTCATCGTTGCGGCTGGAGCTTCTGCACGAAGAGGTCACCATTGTTACATGCTCAAACGCGTAAAGCACCAGATCCACCGCATCGGAGAAAATCATCATGAAGCGGGTCATGCTCGGGTCAGTAATGGTCAGAGGTTTGCCAGAACGCATTGTTCGATTAACAGCGGAATCCACTGACCCCCTCGAAGCATTACGTTACCGTAACGAGTACCCCGATGCCGTGTA

**Figure S4-2**. DNA sequencing to confirm deletion mutant *∆4238.*


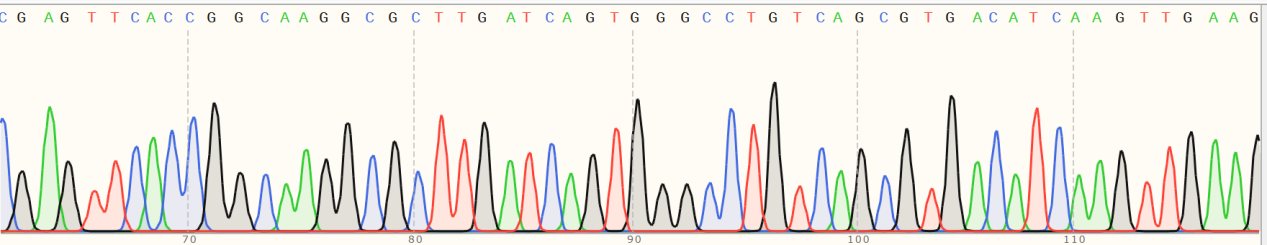


>*∆exoU*

GTGGCTGGGAATGACGCGGCTTCGTGGCTACCCGCAGGCGATCCGCACCGACCAGGGCCCCGAGTTCACCGGCAAGGCGCTTGATCAGTGGGCCTGTCAGCGTGACATCAAGTTGAAGCTGATTCAGCCTGGCCAGCCCACGCAGAGCGCCTTCATCGAGTCATTCAACGGCAAGTTCCGGGGCGAATGCCTCAATGAGCACTGCTCGCTGGTCGAAGCCAGAATCCGTATCGCGGCTTGGCGGGATTACAACGAGCACCGACCACACAGCGCCATTGGCAATCTCTCCCCGGCAGAGCTTGCTGCGAAGTGGCGAACCAACCAGCAGCAGCTGAAGCGGGAAAAGTTGATATCAACCCCATAGCCTACTAACTAGGCAGCGGTACTAAAACTGGGGGCAGGCCAGTCCGAACTGATAAAAAAACGTCCGAAACTGGCAAAACCTTATGGCCGGTTTTCCTAATATTTGCTCCGAACCCTCGGTATTTCAAGGAGCGAACTTGATACATGGCTGGCACAGTGGGGCTTGAGACTTCCCTCGAGCAACGATGCCACGTTGCGGCTGCAACCGGCAGAGGGACCGGAACTGGTTATGGAGCGCCTCGAGGGCGGTTGGCTTTTCGTCGTCGAGTTGGGACTTGTGCCTTCAGGGTTACCGCTGGGTGTGATCTTGCAATTGTTACAAGTGAACTCTCCATTCTCATCCTTGGCACCGGTGAAACTTGCGGCGGACGATGCCGGTAGACTTGTGCTCTGGGCTGAGGCACGTGATGGCTTTGACGATGTGGATGCACTGAACCGCTTGCACGATAGGCTGCGGGAAGGACATTCACGATTAGTGCCATTGCTAGAGCCCACGGGTGAGTTGGTTCCAGCTCAGATACAAACCAGG

**Figure S4-3**. DNA sequencing to confirm deletion mutant *∆exoU.*


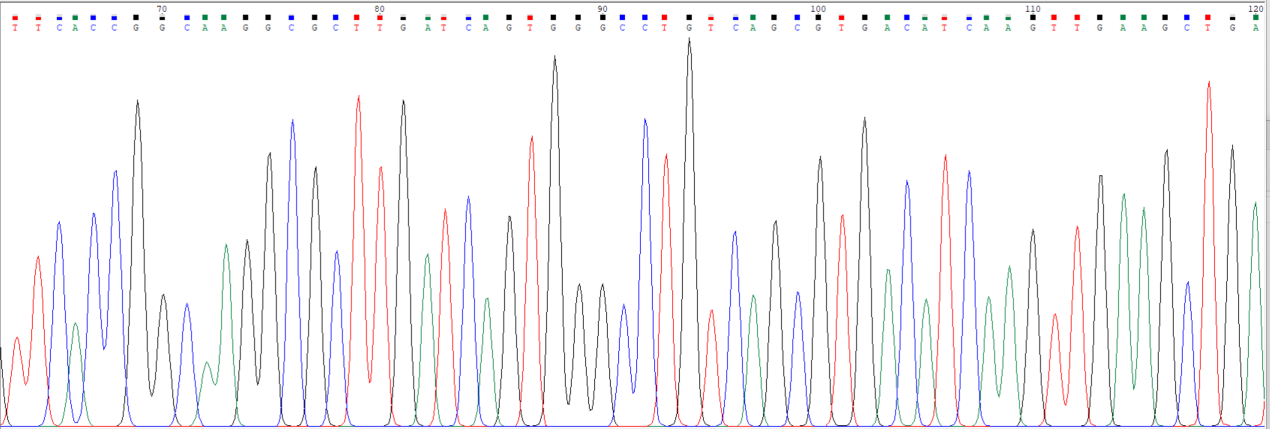


>*∆exoU (exoU)*

CGTTAGAGATGGCGCGATTTCGTGGCTACCCGCAGGCGATCCGCACCGACCAGGGCCCCGAGTTCACCGGCAAGGCGCTTGATCAGTGGGCCTGTCAGCGTGACATCAAGTTGAAGCTGATTCAGCCTGGCCAGCCCACGCAGAGCGCCTTCATCGAGTCATTCAACGGCAAGTTCCGGGGCGAATGCCTCAATGAGCACTGCTCGCTGGTCGAAGCCAGAATCCGTATCGCGGCTTGGCGGGATTACAACGAGCACCGACCACACAGCGCCATTGGCAATCTCTCCCCGGCAGAGCTTGCTGCGAAGTGGCGAACCAACCAGCAGCAGCTGAAGCGGGAAAAGTTGATATCAACCCCATAGCCTACTAACTAGGCAGCGGTACTAAAACTGGGGGCAGGCCAGTCCGAACTGATAAAAAAACGTCCGAAACTGGCAAAACCTTATGGCCGGTTTTCCTAATATTTGCTCCGAACCCTCGGTATTTCAAGGAGCGAACATGCATATCCAATCGTTGGGGGCTACTGCCTCCTCGCTGAATCAGGAGCCTGTCGAAACCCCGTCGCAGGCAGCGCATAAGTCCGCCAGCTTGCGTCAGGAACCTTCAGGGCAAGGTCTCGGGGTTGCCCTAAAGAGCACGCCGGGAATACTTTCCGGGAAGTTGCCGGAAAGCGTTAGCGACGTGCGTTTCAGCAGTCCCCAAGGGCAAGGGGAGTCCCGTACTCTGACTGACTCGGCAGGGCCGCGGCAGATCACTCTGCGCCAGTTTGAGAACGGAGTCACCGAGCTACAGCTCAGTCGGCCACCATTGACCAGTCTGGTCCTAAGCGGCGGTGGTGCCAAAGGTGCGGCATACCCGGGAGCAATGCTGGCGCTAGAAGAGAAAGGCATGCTCGATGGCATCCGCAGCATGTCCGGTTCGTCCGCTGGCGGCATCACCGCCGCCCTTTTGGCCTCAGGTATGAGCCCGGCGGCGTTCAAGACCCTTTCCGACAAGATGGATCTTATTTCGCTGCTCGACAGCTCGAACAAGAAGCTGAAGTTGTTCAACCCATTA

**Figure S4-4**. DNA sequencing to confirm the complementation strain *exoU (exoU).*
